# Supplementary figures and images for: Suppression of migration and invasion by taraxerol in the triple-negative breast cancer cell line MDA-MB-231 via the ERK/Slug axis
Source: PLoS One. 2023 Sep 26;18(9):e0291693. doi: 10.1371/journal.pone.0291693 (PMC10522031; doi:10.1371/journal.pone.0291693)

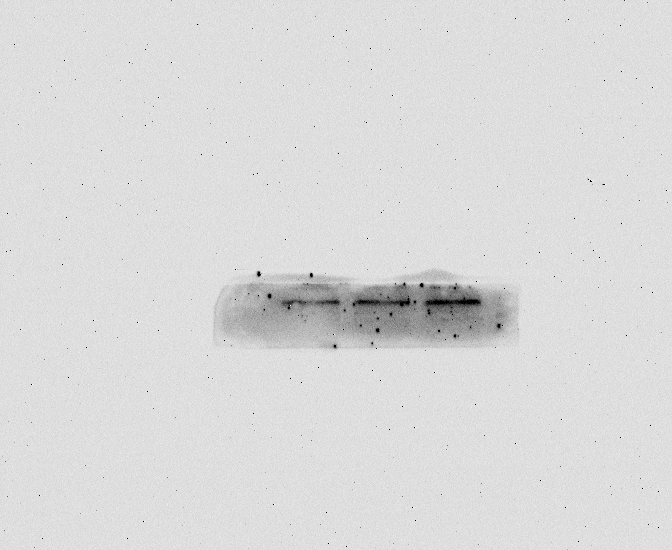

Supplement: S1 Raw images — (ZIP) [file pone.0291693.s001.zip › S1_raw_images/fig.1 F E-cadherin exposure.tif]

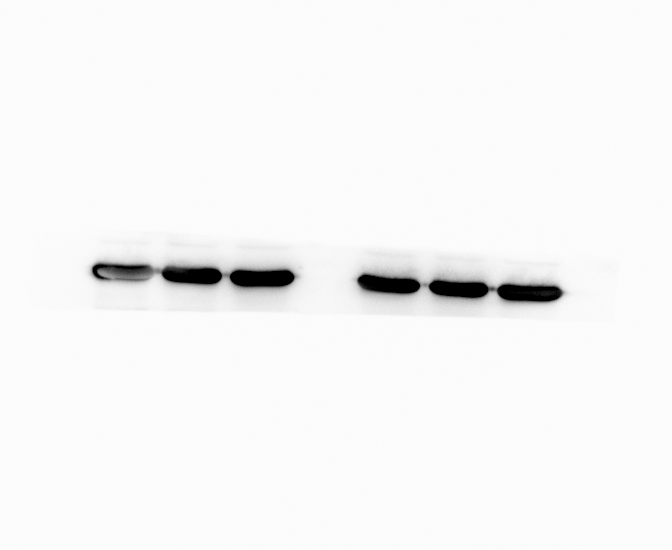

Supplement: S1 Raw images — (ZIP) [file pone.0291693.s001.zip › S1_raw_images/fig.1 F GAPDH(right) exposure.tif]

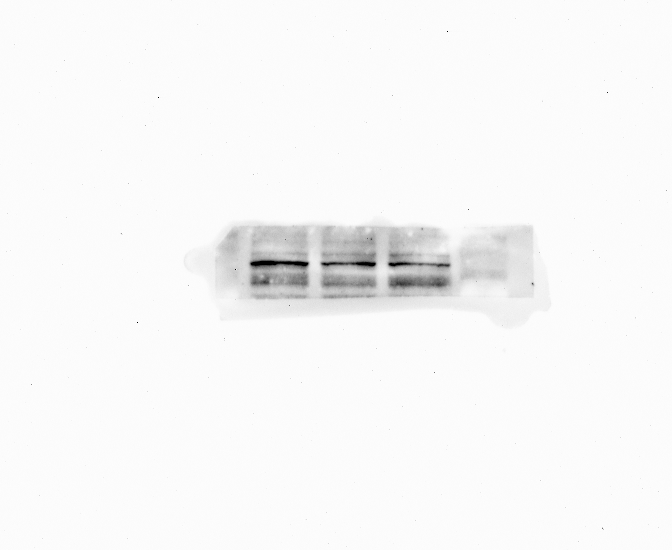

Supplement: S1 Raw images — (ZIP) [file pone.0291693.s001.zip › S1_raw_images/fig.1 F N-cadherin exposure.tif]

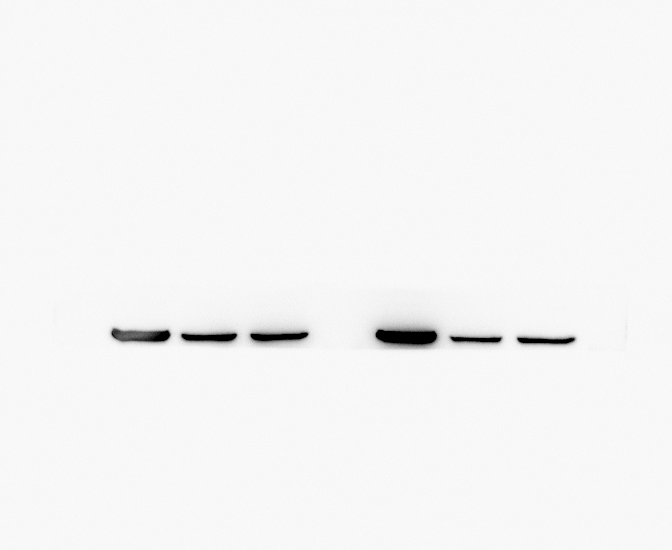

Supplement: S1 Raw images — (ZIP) [file pone.0291693.s001.zip › S1_raw_images/fig.1 F Vimentin(right) exposure.tif]

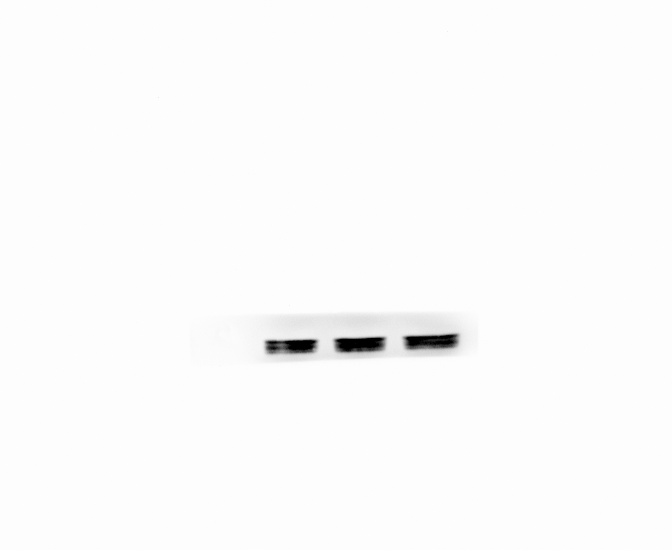

Supplement: S1 Raw images — (ZIP) [file pone.0291693.s001.zip › S1_raw_images/fig.3 B ERK exposure.tif]

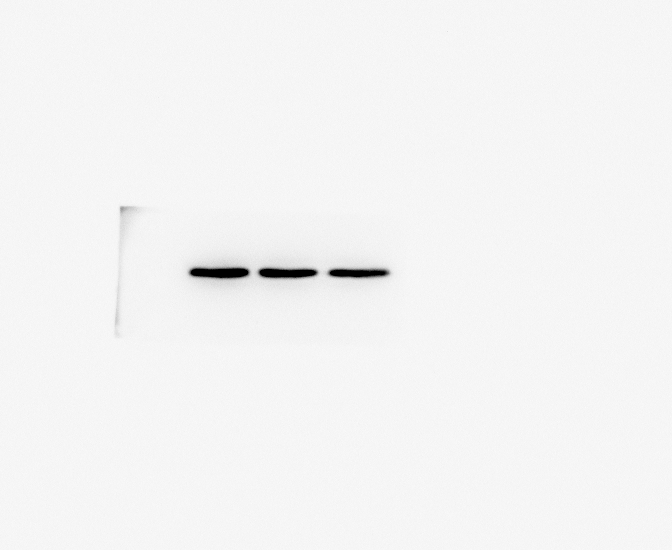

Supplement: S1 Raw images — (ZIP) [file pone.0291693.s001.zip › S1_raw_images/fig.3 B GAPDH exposure.tif]

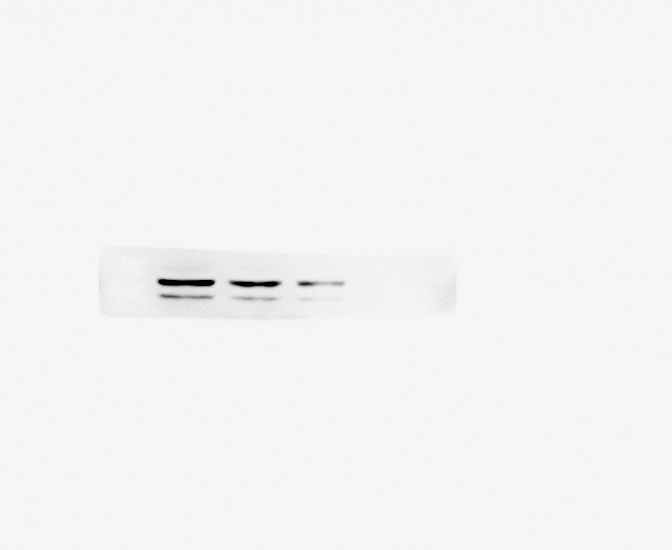

Supplement: S1 Raw images — (ZIP) [file pone.0291693.s001.zip › S1_raw_images/fig.3 B Slug exposure.tif]

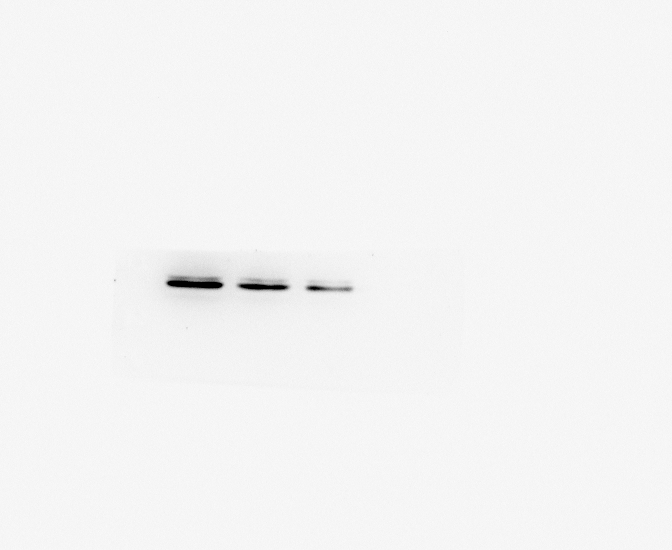

Supplement: S1 Raw images — (ZIP) [file pone.0291693.s001.zip › S1_raw_images/fig.3 B p-ERK exposure.tif]

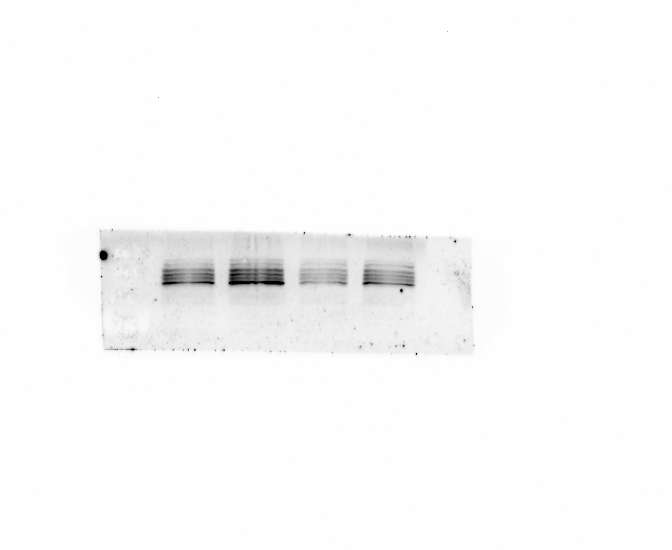

Supplement: S1 Raw images — (ZIP) [file pone.0291693.s001.zip › S1_raw_images/fig.4 E E-cadherin exposure.tif]

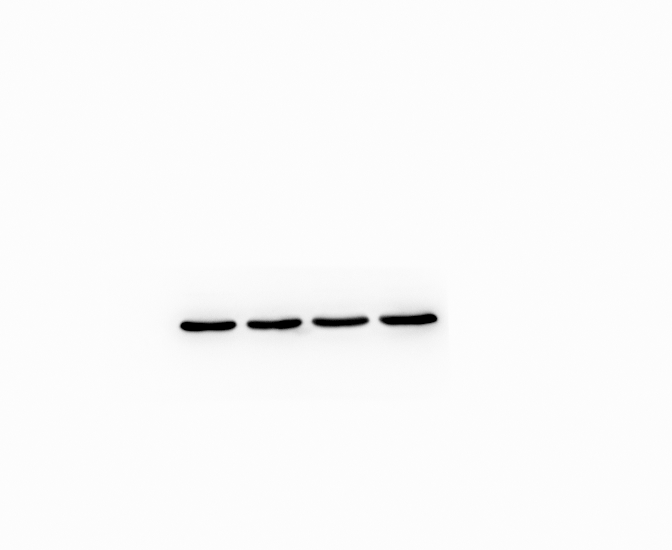

Supplement: S1 Raw images — (ZIP) [file pone.0291693.s001.zip › S1_raw_images/fig.4 E GAPDH exposure.tif]

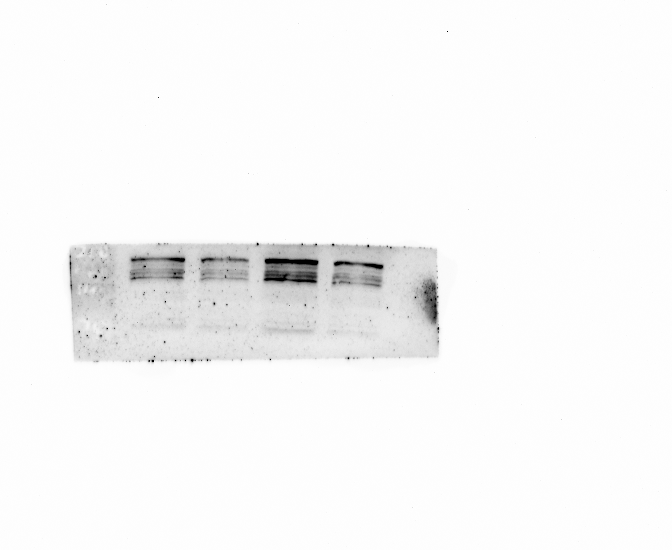

Supplement: S1 Raw images — (ZIP) [file pone.0291693.s001.zip › S1_raw_images/fig.4 E N-cadherin exposure.tif]

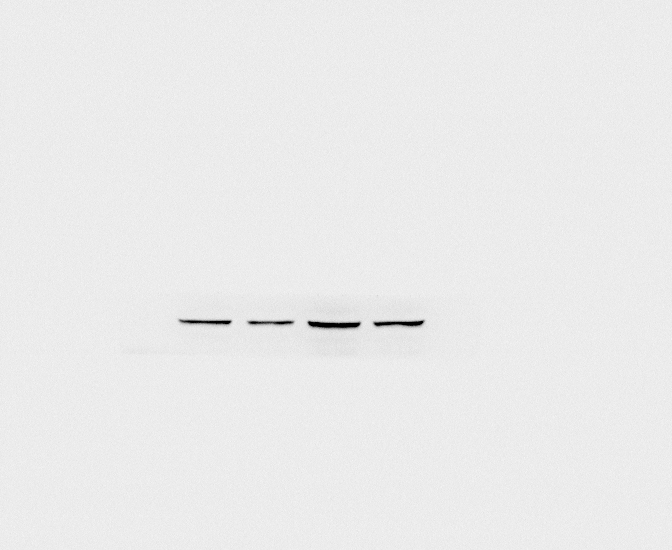

Supplement: S1 Raw images — (ZIP) [file pone.0291693.s001.zip › S1_raw_images/fig.4 E Vimentin exposure.tif]

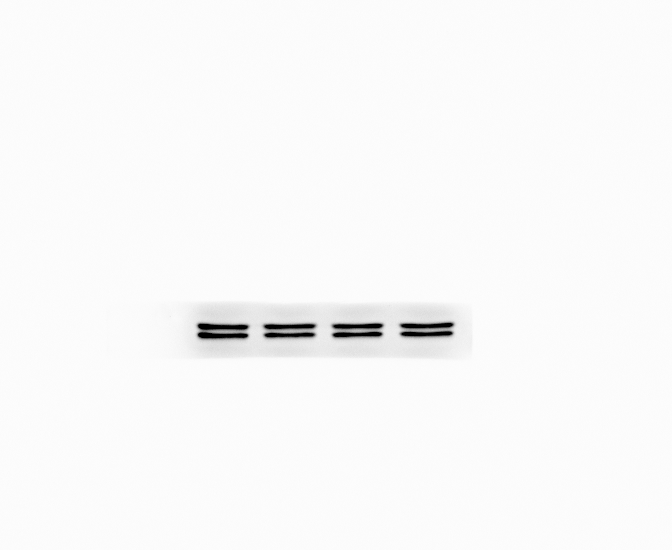

Supplement: S1 Raw images — (ZIP) [file pone.0291693.s001.zip › S1_raw_images/fig.4 G ERK exposure.tif]

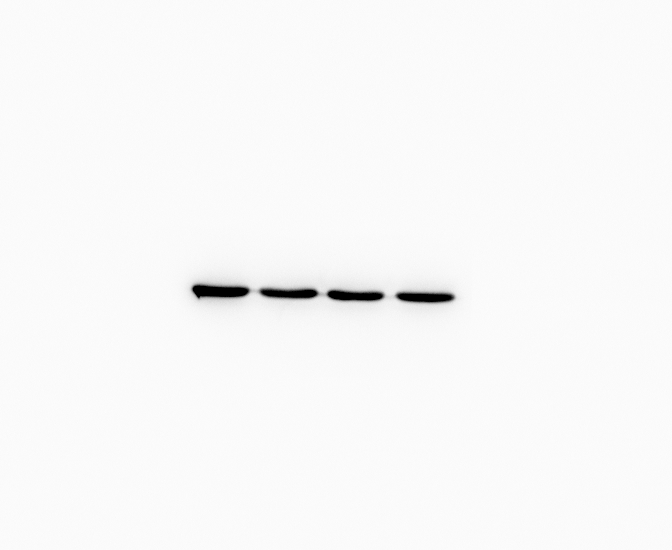

Supplement: S1 Raw images — (ZIP) [file pone.0291693.s001.zip › S1_raw_images/fig.4 G GAPDH exposure.tif]

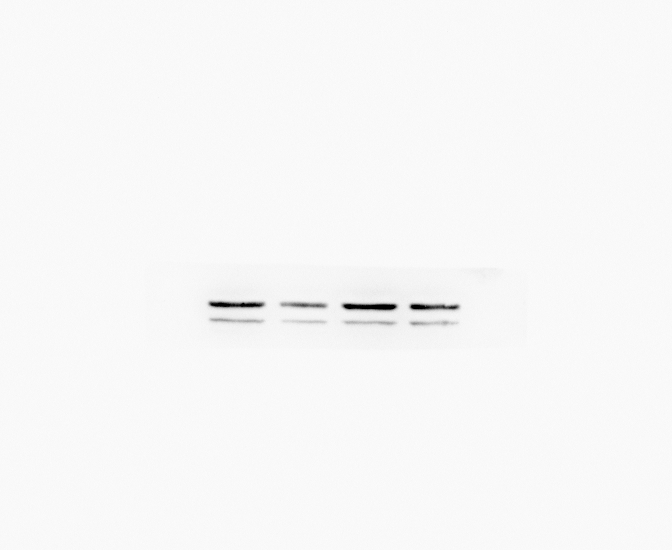

Supplement: S1 Raw images — (ZIP) [file pone.0291693.s001.zip › S1_raw_images/fig.4 G Slug exposure.tif]

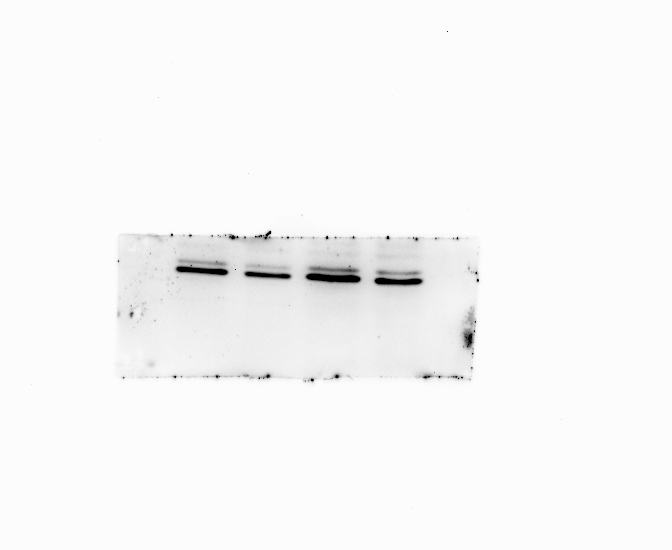

Supplement: S1 Raw images — (ZIP) [file pone.0291693.s001.zip › S1_raw_images/fig.4 G p-ERK exposure.tif]

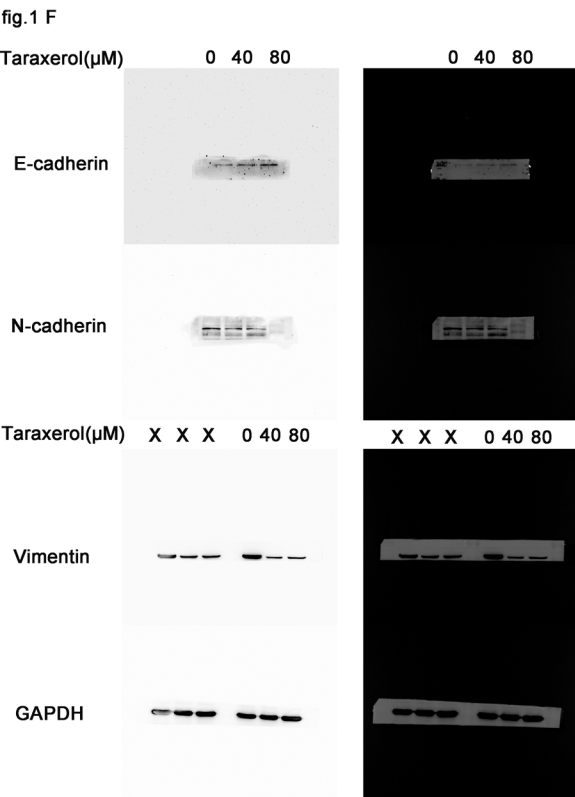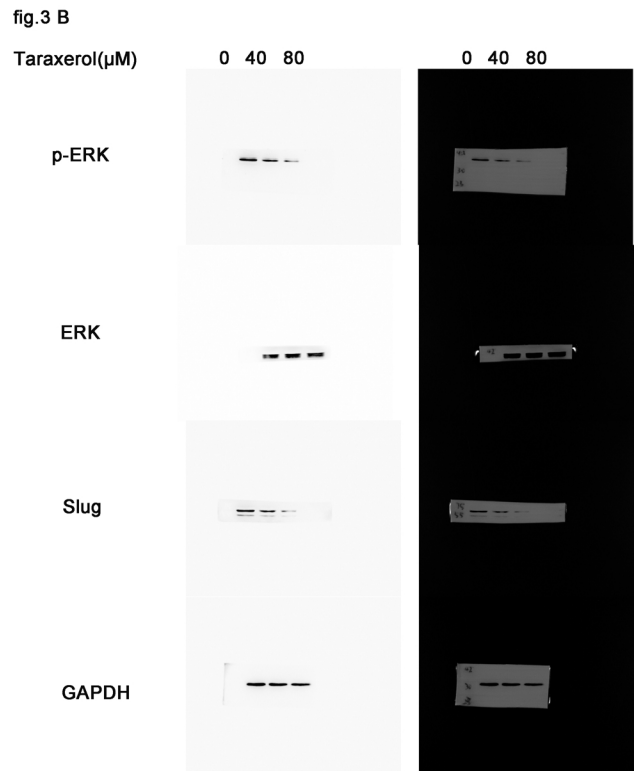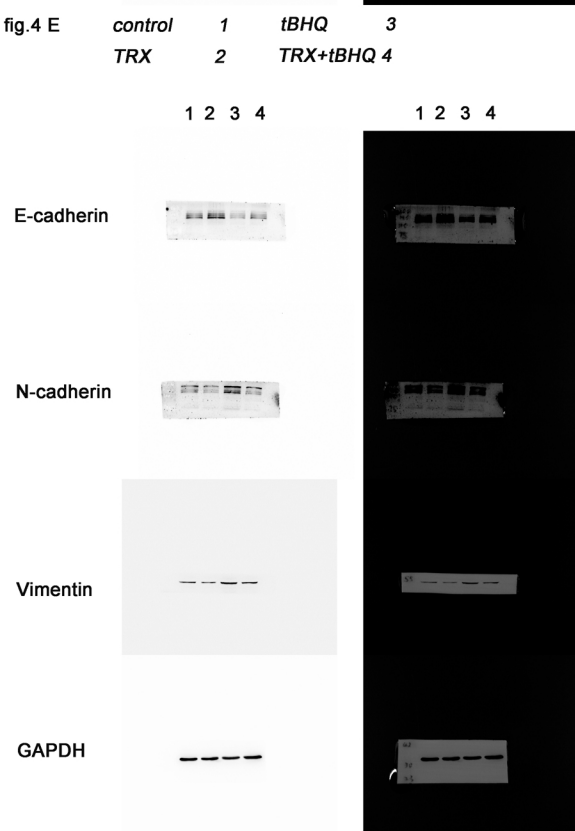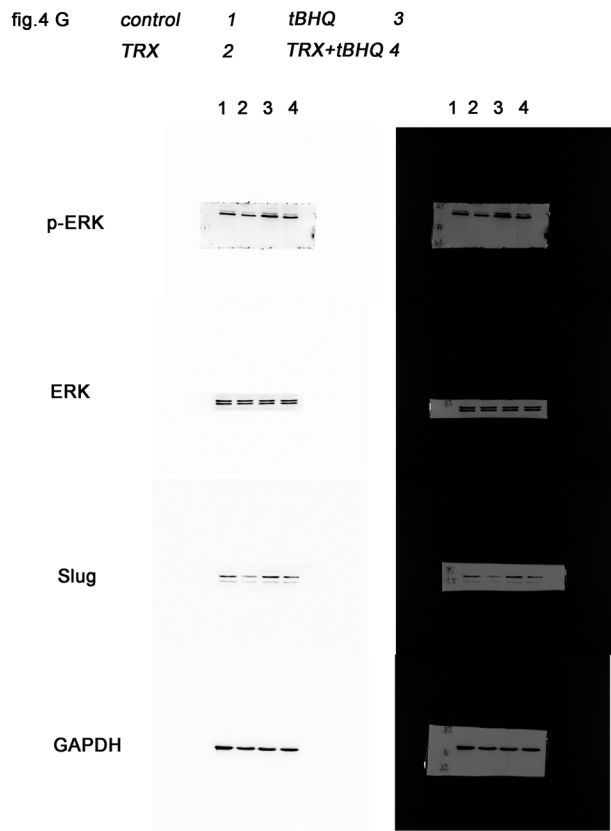

Supplement: S1 Raw images — (ZIP) [file pone.0291693.s001.zip › S1_raw_images/orignal images.pdf]
