# Supplementary material for: Suppression of migration and invasion by taraxerol in the triple-negative breast cancer cell line MDA-MB-231 via the ERK/Slug axis
Source: PLoS One. 2023 Sep 26;18(9):e0291693. doi: 10.1371/journal.pone.0291693 (PMC10522031; doi:10.1371/journal.pone.0291693)
Supplement: Attachment — Submitted filename: Response to Reviewers.docx [file pone.0291693.s002.docx]

Dear Editor:

Thank you for your and the reviewers’ comments on our manuscript entitled “Suppression of migration and invasion by taraxerol in the triple-negative breast cancer cell line MDA-MB-231 via the ERK/Slug axis”. (EMID: f1702d110dff026b). Our deepest gratitude goes to the anonymous reviewers for their careful work and thoughtful suggestions that have helped improve this manuscript substantially. Meanwhile, thank you for giving us a chance to revise and resubmit our manuscript.

Based on these comments, we have made careful modifications again. All changes made to the manuscript are marked in red. These changes will not influence the content and framework of the manuscript. We hope that these revisions are satisfactory and the revised manuscript will be acceptable for publication.

Below you will find our point-by-point responses to your comments/questions. Once again, thank you for your comments and suggestions.

Sincerely,

XIA Yu-ting, ZHANG Yu-qin, CHEN Lu, MIN Liang-liang, HUANG Da, ZHANG Yu-lu, LI Cong, LI Zhi-hua*

*Corresponding author. E-mail address: [huazhili0802@163.com](mailto:huazhili0802@163.com)

**Academic Editor comments:**

1. Please express MTT assay results as viable cells (% of control), change the figure 1a accordingly, and add information about limitations of MTT assay in the discussion part. You can find relevant information here -DOI: 10.1097/CAD.0000000000001131.

Response: Thank you for your advice. We wrongly express MTT assay results as OD values, so we have modified the Figure 1a and add ‘MTT assay relies on cell number to determine cell viability, while the experimental results are greatly influenced by the seeding density. Thus, it is necessary to support this conclusion through other experiments.’ in the discussion part. The Figure 1a is as follows:

2. Please perform molecular dynamic simulation to validate docking results.

Response: Thank you for your advice. In this manuscript, we predicted that MAPK3 was the most potential target of TRX in treatment of TNBC via network pharmacology. In order to enhance the persuasiveness of this results, we used the molecular docking to simulate the binding of ligand 2ZOQ which was the crystal structure of MAPK3 to receptor TRX. It was found that 2ZOQ and TRX could stably bind with hydrogen bonds, hydrophobic interactions and salts bridges.

At the same time, we also understood the limitations of molecular docking and the necessary of molecular dynamic to determine whether ligand and receptor can stably bind in practical environments. But for this research, we were more concerned about whether TRX regulate the expression of MAPK3 in MDA-MB-231 cells than how it regulates it. Thus, we chose to directly verify the prediction results from network pharmacology and molecular docking through WB experiment rather than molecular dynamics. As shown in fig. 4B, TRX significantly suppressed the phosphorylation level of ERK. Therefore, we concluded that MAPK3 was the target of TRX in treatment of TNBC.

3. Manuscript needs significant English improvement.

Response: We apologized for the poor language of our manuscript. We worked on this manuscript for a long time and the repeated addition and removal of sentences and sections obviously led to poor readability. We have involved native English-speaking editors at AJE for language corrections. We really hope that the language level has been substantially improved.

**Reviewer #1**:

This article "Suppression of migration and invasion by taraxerol in the triple-negative breast cancer cell line MDA-MB-231 via the ERK/Slug axis" is interesting, but I feel it's not a completed work. Authors focused on Slug only but there are important transcription factors (e.g., Twist, Snail, Zeb etc), especially Twist1 is a master regulator of cadherins as well as a key factor in EMT associated metastasis. What's the role of taraxerol on Twist in MDA-MB-231? Also, author claims that taraxerol suppress cell migration and invasion by ERK/Slug axis. Is it direct suppression or indirect? May be, it is the secondary effect of other mechanism? May be reporter assay could clarify this. To consider this paper for publishing, I think these issues need to be clarified.

Response: Thank you for your advice. In this study, KEGG enrichment analysis suggested that TRX may inhibit the migration and invasion of MDA-MB-231 cell via ERK/Slug axis, and there were no other EMT related transcription factors in adhesion junction pathway, so we only verified ERK/Slug axis in protein expression level. Furthermore, we acknowledged that had not yet clarified whether this suppression was direct or indirect. However, the mechanism of this study is still in the exploratory stage, and further clarification on these issues will be provided in future research.

**Reviewer #2:**

1. The manuscript is lacking specific information about MDA-MB-231 cells. It is recommended to provide details about MDA-MB-231 cells in their initial mention within the introduction of the manuscript.

Response: Thank you for your feedback. We have added specific information for MDA-MB-231 cells. The sentence is as follows: MDA-MB-231 human TNBC cell line was obtained from the Key Laboratory of Breast Diseases in Jiangxi Province and cultured in high glucose DMEM with 10% fetal bovine serum (FBS).

2. In Figure 1b, the cell density at 0 hours appears inconsistent across all the wells. Please observe the 80 µM TRX-treated well at 0 hours and the 0 µM TRX-treated treated well at 0 hours, as clear disparities in cell concentration are evident. Are there any representative photographs available that show equal cell density in all the wells at the initial time point?

Response: Thank you for your careful review. In order to control variables in the wound healing assay, we seeded the same number of cells and cultured for same time before scratching the bottom of the plate. However, sometimes the lack of cell uniformity leaded to differences in cell concentration. We have replaced some of the photographs as much as possible to ensure consistent initial cell density. The Figure 1b is as follows:

3. The authors' bioinformatics approach in identifying MAPK3 as the target of TRX is quite fascinating. However, figure 2 is not acceptable to me because of its poor resolution. I was unable to verify the details in the figure due to its blurry appearance. Kindly submit a higher-resolution version of Figure 2 for review.

Response: We apologized for the poor resolution of figure 2 and have replaced it with in 600 dpi. The Figure 2 is as follows:

4. Once again, Figure 3a is deemed unacceptable due to its blurry appearance. Kindly provide an improved figure with a higher resolution.

Response: We apologized for the poor resolution of Figure 3a once again and have replaced it with in 600 dpi. The Figure 3a is as follows:

5. In the text of the Results section, it is mentioned that the Western blot results indicate a significant suppression of p-ERK and Slug expression by TRX in a time- and concentration-dependent manner. However, upon examining Figure 3c, it is apparent that the authors only presented a concentration-dependent effect of TRX, and no demonstration of a time-dependent effect is evident.

Response: Thank you for your careful review. We did only present a concentration-dependent effect of TRX, and we have corrected this clerical error. The sentence is as follows: The Western blot results showed that TRX significantly suppressed the expression of p-ERK and Slug in a concentration-dependent manner.

6. The mechanistic details of TRX-induced anti-migratory and anti-invasive effects on MDA-MB-231 cells have been supported by Western blotting data. However, to enhance the strength of the findings and improve the manuscript's quality, the authors could consider including immunofluorescence staining or any other supplementary data. Incorporating imaging data would provide additional support and bolster the validity of their conclusions. It is recommended that the authors consider this suggestion.

Response: Thank you for your feedback. We acknowledged the limitations of Western blotting data. Therefore, we have supplemented the immunofluorescence experiment according to reviewer #2 comments. The results shown in figure 4i indicated that TRX could decrease the expression of ERK phosphorylation and Slug. Thus, we believed that the suppression of migration and invasion by TRX in MDA-MB-231 cells via the ERK/Slug axis.

**Reviewer #3:**

Introduction:

1. No space between the end of sentence and the references in the introduction section. This has been observed throughout the manuscript.

Response: Thank you for your careful review. We have added space between the end of sentence and references throughout the manuscript.

2. Authors need to elaborate the meaning of TNBC and TRX before they start using it in the sentence.

Response: Thank you for your feedback. We have elaborated the meaning of TRX on the second line of Abstract. And we have added the meaning of TNBC on Keywords.

Materials and methods:

1. Authors need to mention what kind of cell line MDA-MB-231 is?

Response: Thank you for your feedback. We have added specific information for MDA-MB-231 cells. The sentence is as follows: MDA-MB-231 human TNBC cell line was obtained from the Key Laboratory of Breast Diseases in Jiangxi Province and cultured in high glucose DMEM with 10% fetal bovine serum (FBS).

2. MTT assay: The cell count should be written as 4 x 103 cells.

Response: Thank you for your careful review. We have modified 4000 cells to 4 x 10^3^ cells.

3. Wound healing assay: Mention the model of microscope and what kind of microscope was used to capture images.

Response: We have added the model and kind of microscope is as follows: The wounds were photographed by microscope (OLYMPUS CKX53, 10x) after culture for 0, 24, and 48h, and the wound areas were measured by ImageJ software.

4. Authors need to mention the version of any bioinformatics or statistical software used throughout the manuscript. For example: GraphPad Prism, ImageJ software etc.

Response: Thanks for your advice. In this manuscript, we used ImageJ 1.53k software to measure the wound areas in wound healing assay and analyzed the gray values in western blot. The above information had been mentioned in materials and methods. We used GraphPad Prism 8.0.1 software to statistically analyze data and draw statistical charts, the sentence is as follows: All data statistical analysis and charts were provided by GraphPad Prism software, and the results are presented as the mean and standard deviation (mean ± SD).

5. Provide catalogue details for reagents such as Matrigel.

Response: Thanks for your advice. It was our negligence to omit some reagents information. Transwell insert and Matrigel were purchased from Corning (New York, USA). The detailed information of other reagents is shown in the Table 1.

Table 1 Reagent and sources

| Reagent Name | Manufacturer | Lot Number |
| --- | --- | --- |
| Dimethyl sulfoxide | Macklin, China | C12804515 |
| Dulbecco’s Modified Eagle Medium | Solarbio, China | 20220921 |
| RPMI-1640 Medium | Thermo Fisher, USA | 8120522 |
| Fetal bovine serum | TIANHANG, China | 18040502 |
| Penicillin-streptomycin liquid | Solarbio, China | 20210717 |
| Phosphate buffer saline | Solarbio, China | 20220104 |
| MTT cell proliferation and cytotoxicity assay kit | Solarbio, China | 715F0510 |
| Insert 24w 8μm transparent | CORING, USA | 353097 |
| Matrigel matrix | CORING, USA | 356234 |
| Crystal violet ammonium oxalate solution | Solarbio, China | 20200924 |
| Paraformaldehyde | LABGIC, China | 70071800 |
| RIPA buffer | Solarbio, China | 20221126 |
| BCA protein assay kit | Solarbio, China | 20220928 |
| SDS-PAGE loading buffer, 5x | Beyotime, China | P0015L |
| SDA-PAGE gel kit | Solarbio, China | 20220919 |
| Prestained protein marker Ⅱ | Servicebio, China | MPC2209031 |
| Tris-glycine running buffer, 5x | Solarbio, China | 20220406 |
| Methanol | XILONG, China | B2203211 |
| WB transfer buffer, 10x | Solarbio, China | 20220810 |
| TBS, 20x | Solarbio, China | 20221023 |
| Tween-20 | Solarbio, China | 223Y013 |
| Non-fat powdered milk | Solarbio, China | 107B055 |
| Ultrasensitive ECL detection kit | Proteintech, China | 20031305 |

6. Western blotting: Authors need to rewrite the last sentence as the photographs cannot be taken by ECL rather the bands were developed using ECL reagents.

Response: Thank you for your careful review. We have rewritten the last sentence of Western blot is as follows: The protein bands were developed by using hypersensitive enhanced chemiluminescence (ECL), and the gray values were analyzed by ImageJ software.

7. Network Pharmacology: Why the cut-off of probability>0.1 was selected for TRX target?

Response: Thank you for your feedback. There were 100 targets predicted by SwissTargetPrediction database, including 46 targets with probability=0 and 54 targets with probability＞0.1. Considering that the targets with probability=0 are meaningless, Thus, we selected the 54 targets with probability＞0.1 as TRX targets.

8. Network pharmacology: Authors need to give a clear explanation about how the results were visualized by bioinformatics in the last sentence.

Response: Thanks for your advice. After GO and KEGG enrichment analysis, the results were visualized through Micro Bioinformatics, an online bioinformatics analysis, visualization cloud platform. Thus, we obtained two histograms and the Sankey bubble diagram. The last sentence is as follows: Meanwhile, GO and KEGG enrichment analyses were carried out visualized through Micro Bioinformatics, an online bioinformatics analysis, visualization cloud platform.

Results:

1. Cell viability: Authors should plot the graphs as cell viability (%) since OD value hardly makes any sense here.

Response: Thank you for your advice. We wrongly express MTT assay results as OD values, so we have modified the Figure 1a and add ‘MTT assay relies on cell number to determine cell viability. The Figure 1a is as follows:

3. Figure 1C: Authors need to have one more set of bars with 0h at 0 mM, 40 mM and 80 mM. The statistical analysis for the treated groups should be based on this set.

Response: Thank you for your careful review. We have added another set of bars with 0h at different concentrations and the Figure 1c is as follows:

4. Figure 1C: Authors need to be elaborative in writing down the percentage results in the result section. When it says, decreased/ increased significantly readers need to know to which extent the differences were observed.

Response: Thank you for your advice. We have added the percentage results in the result section. The sentence is as follow: Compared to control area, the wound area of 0mM, 40mM and 80mM were 69.6%, 83.2% and 89.7% at 24h, the wound area of 0mM, 40mM and 80mM were 50.4%, 70.5% and 83.8% at 48h.

5. Figure 1D, E: No proper explanation of the results. Authors need to mention clearly which reading (Migration or invasion) was taken from which (upper and lower) well?

Response: Thank you for your careful review. In Figure 1D, the upper wells were photographs of cell migration, and the lower wells were photographs of cell invasion. At the same time, we have added some explanation of the results subsequently: In the cell migration of transwell assay, the cell number of control group was 227 per filed, while the TRX group was 80 per filed. In the cell invasion, the cell number of control and TRX groups were 210 and 97 per filed

6. Figure 2: What are these 20 targets? Include a table as a supplementary file.

Response: Thank you for your advice. The 20 targets were common targets, which belong to both TRX targets and TNBC targets.

| Target | Name | Uniprot ID | Probability* |
| --- | --- | --- | --- |
| AR | Androgen Receptor | P10275 | 0.368134 |
| PTPN1 | Protein-tyrosine phosphatase 1B | P18031 | 0.328034 |
| CYP2C19 | Cytochrome P450 2C19 | P33261 | 0.287795 |
| CYP19A1 | Cytochrome P450 19A1 | P11511 | 0.279775 |
| HMGCR | HMG-CoA reductase | P04035 | 0.151058 |
| ESR1 | Estrogen receptor alpha | P03372 | 0.134972 |
| CYP17A1 | Cytochrome P450 17A1 | P05093 | 0.126929 |
| PTPN2 | T-cell protein-tyrosine phosphatase | P17706 | 0.118883 |
| ESR2 | Estrogen receptor beta | Q92731 | 0.118883 |
| FABP4 | Fatty acid binding protein adipocyte | P15090 | 0.118883 |
| TERT | Telomerase reverse transcriptase | O14746 | 0.118883 |
| FABP3 | Fatty acid binding protein muscle | P05413 | 0.118883 |
| CDC25B | Dual specificity phosphatase Cdc25B | P30305 | 0.118883 |
| PTPRF | Receptor-type tyrosine-protein phosphatase F (LAR) | P10586 | 0.118883 |
| ACP1 | Low molecular weight phosphotyrosine protein phosphatase | P24666 | 0.118883 |
| MAPK3 | MAP kinase ERK1 | P27361 | 0.118883 |
| PTPN11 | Protein-tyrosine phosphatase 2C | Q06124 | 0.118883 |
| NOS2 | Nitric oxide synthase, inducible | P35228 | 0.118883 |
| VDR | Vitamin D receptor | P11473 | 0.118883 |
| ALOX5 | Arachidonate 5-lipoxygenase | P09917 | 0.118883 |

7. Figure 2: As authors have mentioned MAPK3 as the most promising candidate, I would like to see the protein expression level of MAPK3 alongside ERK/p-ERK by western blotting in Figure 3B/4G. Also, if authors are specifically saying that MAPK3 is involved then why they are measuring total p-ERK/ERKs instead of p-ERK1/ERK1? This is contradictory as MAPK1 can also be a major candidate here based on the observation of the western blots in figure 3B and 4G.

Response: Thank you for your feedback. The protein encoded by the MAPK3 gene is ERK1, while the protein encoded by the MAPK1gene is ERK2. In the signaling pathway, the upstream activation pathways and downstream targets of ERK1 and ERK2 are highly similar. Thus, we chose to simultaneously examine the protein expression of ERK1 and ERK2.

The results of Western blot indicated that TRX had the same regulatory effect on ERK1 and ERK2, which indirectly reflects the limitation of network pharmacology. However, the mechanism of this study is still in the exploratory stage, and further clarification on these issues will be provided in future research.

8. Fig 3A: Did author measure the expression of Cadherin in the blots? If not, why the Cadherin in this figure is marked as red? There is no explanation about cadherin in this section. They also need to explain clearly in the figure legend about the scales drawn on the top right side.

Response: Thank you for your advice. In the map04520, the Cadherin referred to E-cadherin. According to fig. 1F, the expression of E-cadherin was upgraded by TRX. In addition, the color of the scale played an important indicating role. Read indicated a positive correlation between gene and TRX, while green indicated a negative correlation.

9. Fig 3B, C: Did authors measure the expression of p-ERK1/ERK1 or both ERKs? If the probing for p-ERK, ERK and GAPDH was done on the same blot after stripping, how does the author confirm that p-ERK and ERK does not cross-react? Authors need to describe how did they quantify p-ERK and ERK level?

Response: Thank you for your feedback. In order to avoid cross-react caused by stripping, we had to repeatedly load the same protein sample on the same gel, so that p-ERK and ERK can be incubated separately. In addition, the marker is shown in the following figure, and the color is displayed at 42kDa and 30kDa. Usually, the PVDF membrane is cut along the upper one-third before incubating the primary antibody, so that GAPDH can be incubated separately.

10. Fig 3B, C: “TRX significantly suppressed the expression of p-ERK and Slug in a time- and concentration-dependent manner”: Wrong statement as authors did not measure the time-dependent effect here.

Response: Thank you for your careful review. We did not measure the time-dependent effect, and we have corrected this clerical error. The sentence is as follows: The Western blot results showed that TRX significantly suppressed the expression of p-ERK and Slug in a concentration-dependent manner.

11. Fig 4A, D: Authors need to give proper explanation for their interpretation?

Response: Thank you for your feedback. We have added the percentage results to illustrate the reverse function of tBHQ. The sentence is as follow: In wound-healing assay, compared to control group, the wound area of TRX group were 91.1% at 24h, 89.6% at 48h. While the wound area of TRX+tBHQ group were 85.6% at 24h, 73.5% at 48h. In the cell migration of transwell assay, the cell number of TRX group was 230 per filed, while the TRX+tBHQ group was 356 per filed. In the cell invasion of transwell assay, the cell number of TRX group was 279 per filed, while the TRX+tBHQ group was 515 per filed.

Discussion:

1. Need reference for this statement: According to the latest data published by the International Agency for Research….

Response: Thank you for your careful review. We have added the reference number 13 for this statement.

2. This sentence doesn’t make sense: Therefore, it is necessary to promote research no innovation drugs.

Response: Thank you for your careful review. We have corrected this clerical error as follows: Therefore, it is necessary to promote research innovation drugs.

3. Is it dedifferentiated? and in dedifferentiated breast cancer cells, it was significantly.

Response: In this paper named E-Cadherin-mediated Cell-Cell adhesion Prevents invasives of human carcinoma cells, its conclusion explicitly mentioned that the selective expression of E-cadherin in differentiated and dedifferentiated cells. At the same time, we can see from Table 1 that the difference is significant. Thus, we believed: in dedifferentiated breast cancer cells, the expression level of E-cadherin was significantly lower than that in differentiated cells.

Figure:

1A: Wrong statement as the OD values cannot be counted by GraphPad software

Response: Thank you for your advice. We have modified the Figure 1a and add ‘MTT assay relies on cell number to determine cell viability, and the Figure 1a is as follows:
